# First detection of *Anaplasma phagocytophilum* and enzootic focus of *Theileria orientalis* in cattle from Bosnia and Herzegovina

**DOI:** 10.3389/fvets.2026.1791187

**Published:** 2026-04-02

**Authors:** Ana Vasić, Goran Vasilić, Andrea Radalj, Isidora Prošić, Jasna M. Kureljušić, Ratko Sukara, Snežana Tomanović, Oliver Stevanović

**Affiliations:** 1 Scientific Institute of Veterinary Medicine of Serbia, Belgrade, Serbia; 2 PI Veterinary Institute of the Republic of Srpska “Dr. Vaso Butozan”, Banja Luka, Bosnia and Herzegovina; 3 Faculty of Veterinary Medicine, University of Belgrade, Belgrade, Serbia; 4 Institute for Medical Research, National Institute of Republic of Serbia, University of Belgrade, Belgrade, Serbia

**Keywords:** *Anaplasma phagocytophilum*, Bosnia and Herzegovina, cattle, *Theileria orientalis*, tick-borne pathogens

## Abstract

*Theileria orientalis* is a tick-borne intraerythrocytic protozoan parasite of cattle causing oriental or benign theileriosis. It has a worldwide distribution and is not considered a zoonotic agent, while the disease symptoms range from subclinical to severe. *Theileria* spp. are very common co-infecting agents with other tick borne pathogens such as *Babesia* spp., *Anaplasma* spp. including zoonotic *Anaplasma phagocytophilum* with rising prominence. This study is an exploratory investigation of the occurrence of *Anaplasma* spp. and other blood parasites in cattle from Gacko and Bileća, Herzegovina. Blood samples from 35 clinically healthy cattle were selected based on veterinary reports of tick infestation and examined microscopically, by PCR (*Babesia/Theileria*; *Anaplasmataceae*) and qPCR (*A. phagocytophilum, A.platys, A.marginale*). Microscopy revealed intraerythrocytic bacterial forms in multiple samples. *A. phagocytophilum* DNA was detected in one and *Babesia/Theileria* DNA in 34 samples. Based on 16S rRNA gene sequencing, the *A. phagocytophilum* isolate was grouped with isolates from Europe, suggesting regional pathogen circulation. *AnkA* gene analysis placed the isolate in European group I, but outside the zoonotic subcluster, indicating a ruminant-associated lineage maintained primarily in wildlife–*Ixodes ricinus* cycles. The 18S rRNA gene sequencing showed subtle regional clustering of *T.orientalis* isolates. This is the first molecular confirmation of *A. phagocytophilum* in cattle from Eastern Herzegovina and identification of possible enzootic foci of *T. orientalis* infection, deeming further investigation into tick vectors and reservoir hosts in the region.

## 1 Introduction

*Anaplasma phagocytophilum* is a cosmopolitan, emerging tick-borne, Gram-negative intracellular bacterium of the *Anaplasma* genus, along with other species *A. platys, A. centrale, A. bovis, A. marginale, A. ovis*, and *A. capra*. It infects domestic animals and humans, leading to significant morbidity, especially in immunocompromised individuals (1–3). The geographic distribution of this bacterium is linked to its primary vector, the hard tick *Ixodes ricinus* (Acari: Ixodidae), and epidemiological studies have shown its presence in different host species across most European countries (3). Reservoir species of *A. phagocytophilum* include large game (such as deer, roe deer, and moose) and rodents (such as mice, shrews, and voles). *Anaplasma phagocytophilum* causes tick-borne fever (also known as pasture fever) in ruminants, characterized by different severity of clinical symptoms ranging from severe in lambs to mild or subclinical in cattle (4). Furthermore, it causes equine granulocytic anaplasmosis (EGA) in horses, and canine granulocytic anaplasmosis (CGA) in dogs. *Anaplasma phagocytophilum* is a zoonotic pathogen causing human granulocytic anaplasmosis (HGA) with flu-like symptoms, fever, chills, headache, myalgia, and leukopenia that can lead to severe complications (2, 5). HGA is often found in areas where Lyme disease is endemic and was by now confirmed in North America, Europe, and Asia, often in co-infections (6).

The distribution of *Anaplasma* species in domestic animals in Bosnia and Herzegovina is still not sufficiently documented, while there is more data available for the distribution of tick vector species in the region and other tick-borne pathogens such as causative agents of Lyme Boreliosis. *Anaplasma phagocytophilum* was detected in ticks (7) and dogs (8, 9) from Bosnia and Herzegovina, but to the best of our knowledge there is no data available for the detections of *A. phagocytophilum* in domestic ruminants. Significant genetic diversity of *A. ovis* was detected in sheep in the Herzegovina region (10), indicating the possible presence of other *Anaplasma* species in the area based on observations of symptoms similar to tick-borne fever by local veterinarians, animal holders, and epidemiological service. Several studies conducted so far indicated the circulation of *A. phagocytophilum* in animal hosts and ticks in the Balkan region. In a study from neighboring Serbia, Vasić et al. found a low seroprevalence of *A. phagocytophilum* in cattle (2.45%; 4/163; 95% CI: 0.96%−6.14%) and no bacterial DNA in the blood of seropositive animals (11). Wild carnivores are recognized reservoirs and potential transmitters of a variety of zoonotic pathogens (12). Another study from Serbia found *A. phagocytophilum* DNA in 0.9% (2/216; 95% CI: 0.26%−3.31%) of golden jackal (*Canis aureus*) spleen samples (13) indicating the role of wild carnivores in the *A. phagocytophilum* transmission cycle in nature. Furthermore, the presence of *A. phagocytophilum* was also detected in ticks, in the regions of Serbia near the border with neighboring Bosnia and Herzegovina (14). In Croatia, *A. phagocytophilum* was found in animal reservoirs and ticks and there have been reported cases of HGA (15, 16).

Oriental theileriosis of cattle is a protozoan disease caused only by the *Theileria orientalis* complex—group *Theileria orientalis/buffeli/sergenti* (17, 18). Depending on the species of *Theileria*, a number of hard ticks of the genera *Amblyomma, Haemaphysalis, Hyalomma* and *Rhipicephalus* can transmit these pathogens (19). Theileriosis caused by *T. orientalis* complex was considered benign and asymptomatic, but clinical cases had been reported from Australia, Japan and New Zeland (20–22). Currently, from 11 genotypes of *T.orientalis* only Ikeda and Chitose are concidered to be pathogenic (18). Cattle infected with high burdens of the Ikeda genotype often become anemic with clinical signs including tachypnoea, lethargy, ataxia, abortion in pregnant animals and mortality in up to 10% of cases. Stressors (e.g., parturition, lactation and transport of infected animals) exacerbate the onset of the disease. The geographical range of *T. orientalis* Ikeda has expanded significantly in the past two decades facilitated by the widespread occurrence of its principal tick vector, *Haemaphysalis longicornis* (23, 24).

*Theileria orientalis* was previously recorded with high prevalence in Sarajevo-Romanija region of Bosnia and Herzegovina (25). Research conducted in Europe also indicates its presence in Italy (26), Greece (27), Spain (28), Portugal (29), Romania (30), Hungary (17), United Kingdom (31), Serbia (11), Croatia (32), and Russia (33). Numerous studies indicate frequent co-infections with *T. orientalis* and *A. marginale* (32, 34, 35) as well as *T.orientalis* and *Babesia* spp. (35–37). According to some studies, single infection with *T.orientalis* or hemoplasmas in cattle is more common than co-infections because of the “interference phenomenon” (38).

Based on previous results on *A. ovis* detection in Eastern Herzegovina and high prevalence of bovine piroplasmosis in the small geographical area of the Sarajevo-Romanija region, this study aimed to determine the presence of *Anaplasma* species (namely *A. phagocytophilum, A. platys*, and *A. marginale* based on epidemiological data in Europe) and *Babesia/Theileria* in cattle in this enzootic area for tick-borne infections in a targeted approach based on previously conducted epidemiological investigation. Furthermore, this study aims to provide the first molecular confirmation and typing using the polymorphic *ankA* locus of *A. phagocytophilum* in cattle from Eastern Herzegovina (39). Sequencing of the **ankA** gene has become a key molecular tool for the phylogenetic grouping of *A. phagocytophilum*, as its high genetic variability enables discrimination of ecotypes and host-associated lineages that cannot be resolved by more conserved markers such as 16S rRNA (40). Our study aims to lay a base for further surveillance of *Anaplasma* species in livestock and their potential impact on animal health and disease transmission, as well as bovine piroplasms and their epidemiological and clinical significance in Bosnia and Herzegovina.

## 2 Materials and methods

### 2.1 Sampling and study area

**Figure 1:**
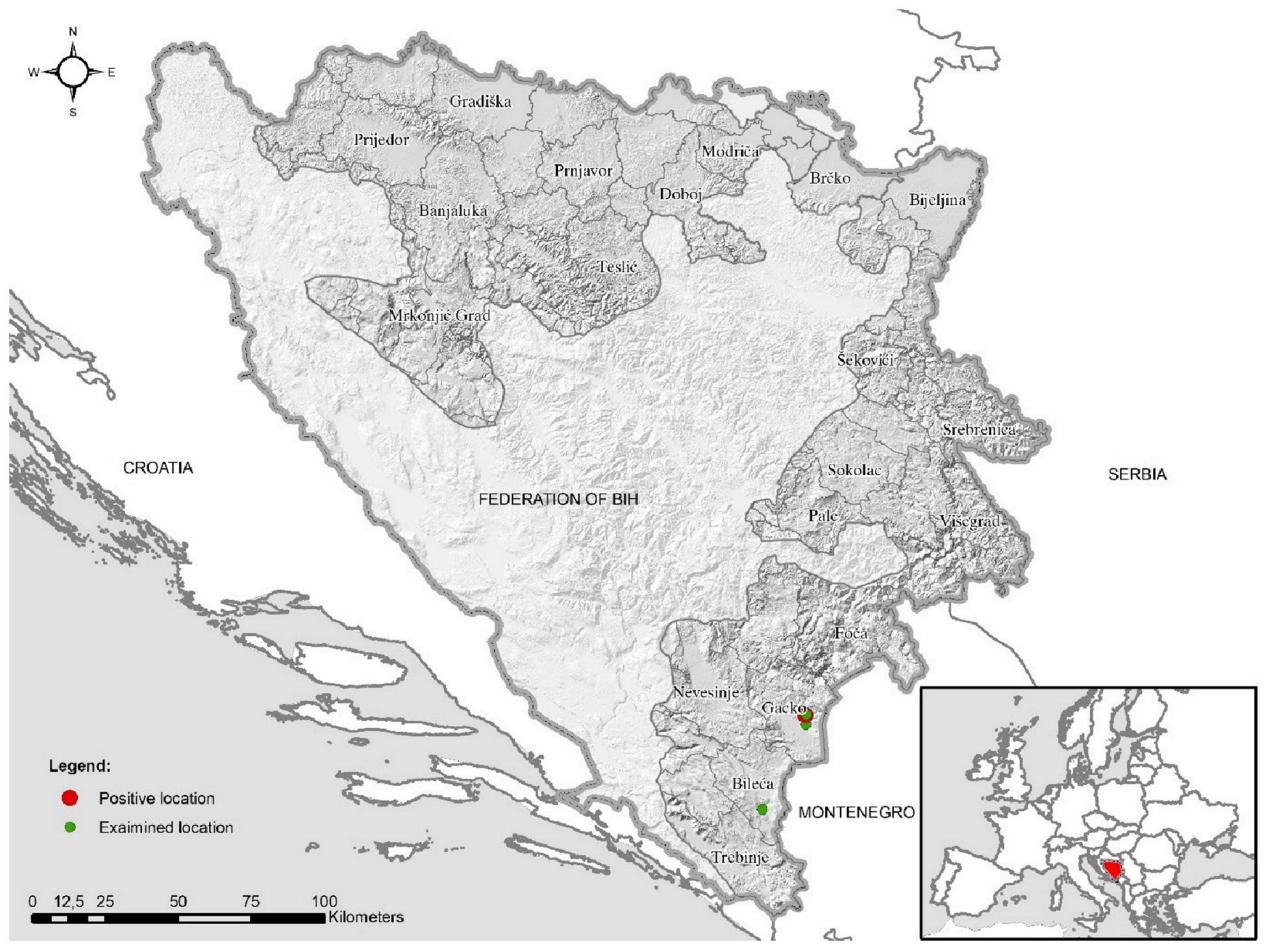
Geographical map of investigated areas of Gacko and Bileća—red circle indicates a positive location for *Anaplasma phagocytophilum* infection.

A total of 35 blood samples from clinically healthy cattle were collected by veterinary practitioners from the municipalities of Gacko (coordinates 43.1667, 18.5333) (*n* = 30) and Bileća (coordinates 42.8765, 18.4297) (*n* = 5) at the beginning of June 2024. Geographically, these epizootiological units belong to the region of Eastern Herzegovina and are characterized by hilly relief and the intersection of Mediterranean and continental–mountainous climates. Sampling was carried out at four locations in the municipality of Gacko and one in the Bileća municipality (Figure 1).

The studied population was selected based on reports by owners and local veterinarians concerning the presence of large numbers of ticks on cattle from February to May, aiming to gain better insight into the diversity of pathogenic species that ticks may transmit to cattle in the area. The study was conducted according to the Law on Animal Protection and Welfare of the Republic of Srpska (“Official Gazette of Republic of Srpska 111/08”). An ethical statement does not apply to this research, as samples were taken during formal examinations by local veterinarians.

Before venipuncture, information was collected on the geographic coordinates of the households, as well as the breed, sex, age, and other important characteristics of the animals, including observations of possible changes in their health status. This information was provided by veterinary practitioners and animal owners. All 35 animals that participated in the research were female, with ages ranging from 2 to 16 years. At the time of sampling (June), no ticks were observed on any of these animals. Blood samples were collected by puncture of the coccygeal vein into EDTA tubes, thin glass slide smears were made and the blood was transported the same day under a cold chain to the Veterinary Institute of the Republic of Srpska “Dr. Vaso Butozan” in Banja Luka, where they were stored at −20 °C until DNA extraction.

### 2.2 Microscopic examination

All collected blood samples were used to prepare thin glass slide smears, which were then air-dried, fixed with methanol, stained with Giemsa, and examined under a light microscope (magnification of 40x to examine the entire surface of the slide and 100x to describe the morphology of the observed structures). The shape of the observed formations was carefully described using terms such as round, pear-shaped, and oval, as well as their number and arrangement (peripheral, subcentral, central) within the blood cells.

### 2.3 DNA extraction

Total DNA nucleic acid extraction from 200 μL of EDTA blood was primarily performed using the commercial IndiSpin Pathogen Kit (Indical Bioscience, Leipzig, Germany) according to the manufacturer's instructions. The DNA extracts were adequately labeled, packed and, as previously described, sent to the Laboratory for Virology and Molecular diagnostics of the Veterinary Institute of the Republic of Srpska “Dr Vaso Butozan” in Banja Luka, after which they were stored at −20 °C until further analysis.

### 2.4 Molecular pathogen detections

The extracted DNA samples were tested by conventional PCR to prove the presence of the *A. marginale* genome, using the specific PCR protocol described by Torina et al., targeting the *msp4* gene (344 bp) (41). The reaction was performed using Himedia Hi—Chrom PCR Master mix (HigenoMB, India) on a Nexus PCR Thermal cycler (Eppendorf, Hamburg, Germany). Electrophoresis of PCR products was performed on a 1.5% agarose gel (100 V, 60 min), pre—stained with Midori green and visualized under UV light.

Aliquots of the tested extracts (30 μl) were packed and sent under a cold chain to the Scientific Institute of Veterinary Medicine of Serbia, where they were stored at −20 °C until further use. First, a 345 bp fragment of the 16S rRNA gene specific to the *Anaplasmataceae* family was targeted according to Parola et al. (42). DNA of *Babesia/Thelieria* was detected by PCR using previously described primers (43). The reactions were carried out with PCR Master Mix 2x (Thermo Fisher Scientific, Waltham, Massachusetts, USA) using an MJ Mini Personal Thermal Cycler (Bio-Rad, Hercules, California, USA), followed by agarose gel electrophoresis stained with Simply Safe™ (EURX, Gdansk, Poland). The amplification products were visualized under UV light. A 6x Orange DNA Loading Dye (Thermo Fisher Scientific, Waltham, Massachusetts, USA) was used for visual monitoring of DNA migration during electrophoresis.

Detection of specific DNA for *A. platys* and *A. phagocytophilum* was performed using primers and probes listed in Table 1. The molecular targets included the *groEL* gene for *A. platys* and the *msp2* gene for *A. phagocytophilum* (44). The reaction was performed using Fast Gene Probe 2x qPCR Universal Mix (Nippon Genetics Europe, Düren, Germany) (primers 10 μM, probe 5 μM) on a MIC (Magnetic Induction Cycler) (Bio Molecular Systems, Brisbane, Australia). The sample identified as *A. phagocytophilum* was further tested using primers specific for the *ankA* gene and prepared for sequencing (45). Each PCR run included adequate positive and negative controls available in each laboratory, and primer sets are listed in Table 1.

**Table 1:** List of pathogens, primers/probe sets and their sequences used in this study.

Pathogen	Primer name	Sequence (55^′^-33^′^)	Reference
*Babesia/Theileria*	BJ BN2	-GTCTTGTAATTGGAATGATGG- -AGTTTATGGTTAGGACTACG-	(42)
*Anaplasmataceae*	EHR 16SD EHR 16SR	-GGTACCYACAGAAGAAGTCC- -TAGCACTCATCGTTTACAGC-	(41)
*Anaplasma marginale*	AmargMSP4Fw AmargMSP4Rev	-CTGAAGGGGGAGTAATGGG- -GGTAATAGCTGCCAGAGATTCC-	(40)
*Anaplasma platys*	An_pla_groEL_F An_pla_groEL_R An_pla_groEL_P	-GCTATGGAAGGCAGTGTTGG- -GTCTTGAAGCGCTCGTAACC- -AATCTCAAGCTCAACCCTGGCACCAC-	(43)
*Anaplasma phagocytophilum*	An_ph_msp2_F An_ph_msp2_R An_ph_msp2_P	-GCTATGGAAGGCAGTGTTGG- -GTCTTGAAGCGCTCGTAACC- -AATCTCAAGCTCAACCCTGGCACCAC-	(43)
*Anaplasma phagocytophilum*	AnkaAP2074s AnkaAP2815a	-GGCAAATGAGGCAAGTAACC- -GCCACTACCCAAGGATGATAG-	(44)

### 2.5 Phylogenetic analysis

The positive PCR product for *Anaplasmataceae* and *Babesia/Theileria* was cut from the gel, purified using Agarose-out DNA purification kit (EURx, Gdansk, Poland) and sequenced in both directions with PCR primers. Consensus sequences were created using MEGA 12 software (46) and were further analyzed using BLAST (http://blast.ncbi.nlm.nih.gov). The pairwise distances were calculated using MEGA 12 software. The evolutionary history for *A. phagocitophylum* was inferred using the Maximum likelihood method and the evolutionary distances were computed using the Kimura 2-parameter method and bootstrap analysis with 1,000 reiterations. In total, 21 nucleotide sequences of *Anaplasma* spp. from GenBank were used for phylogenetic analysis (14 *A. phagocytophilum* strains, 2 *A. marginale*, 3 *A. platys*, and 2 *A. bovis* strains), while *Rickettsia ricketsii* was included as an outgroup. The obtained sequence was deposited to GenBank under the accession number PV056069.

The same sample was subsequently analyzed for the *ankA* gene using specific PCR primers. Maximum Likelihood phylogeny was reconstructed in MEGA 12 using the General Time Reversible substitution model. Rate heterogeneity was modeled with a discrete Gamma distribution (five categories, +G parameter = 2.9617) and a proportion of invariant sites (+I = 0.00%). Codon positions 1st, 2nd, 3rd, and non-coding sites were included, and positions containing gaps or missing data were eliminated using the complete-deletion option, yielding a final alignment of 494 positions. Ninety-one coding nucleotide sequences of *A. phagocytophilum* were compared and grouped according to Rar et al. (39) into 12 established clusters. The resulting *ankA* sequence was deposited in GenBank (accession no. PX255552).

The phylogeny for *T. orientalis* was inferred using the Maximum Likelihood method and Tamura (1992) model of nucleotide substitutions (47). The analytical procedure encompassed 35 nucleotide sequences with 436 positions in the final dataset and included analogous nucleotide *T. orientalis* sequences from GenBank as well as *Babesia bovis* sequence as an outgroup (48). The obtained sequences were deposited to GenBank under the accession numbers PV581906-PV581910.

## 3 Results

### 3.1 Microscopical observation

**Figure 2:**
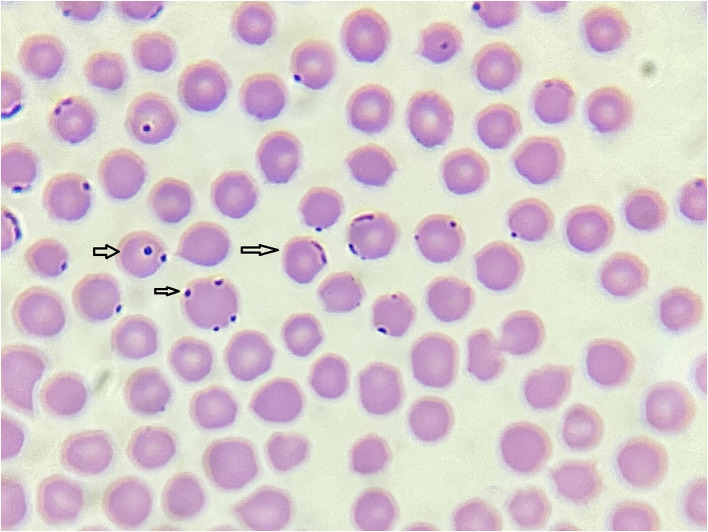
Spherical and coccobacillary bacterial forms in red blood cells (1,000x) (“Dr. Vaso Butozan”, 2024).

**Figure 3:**
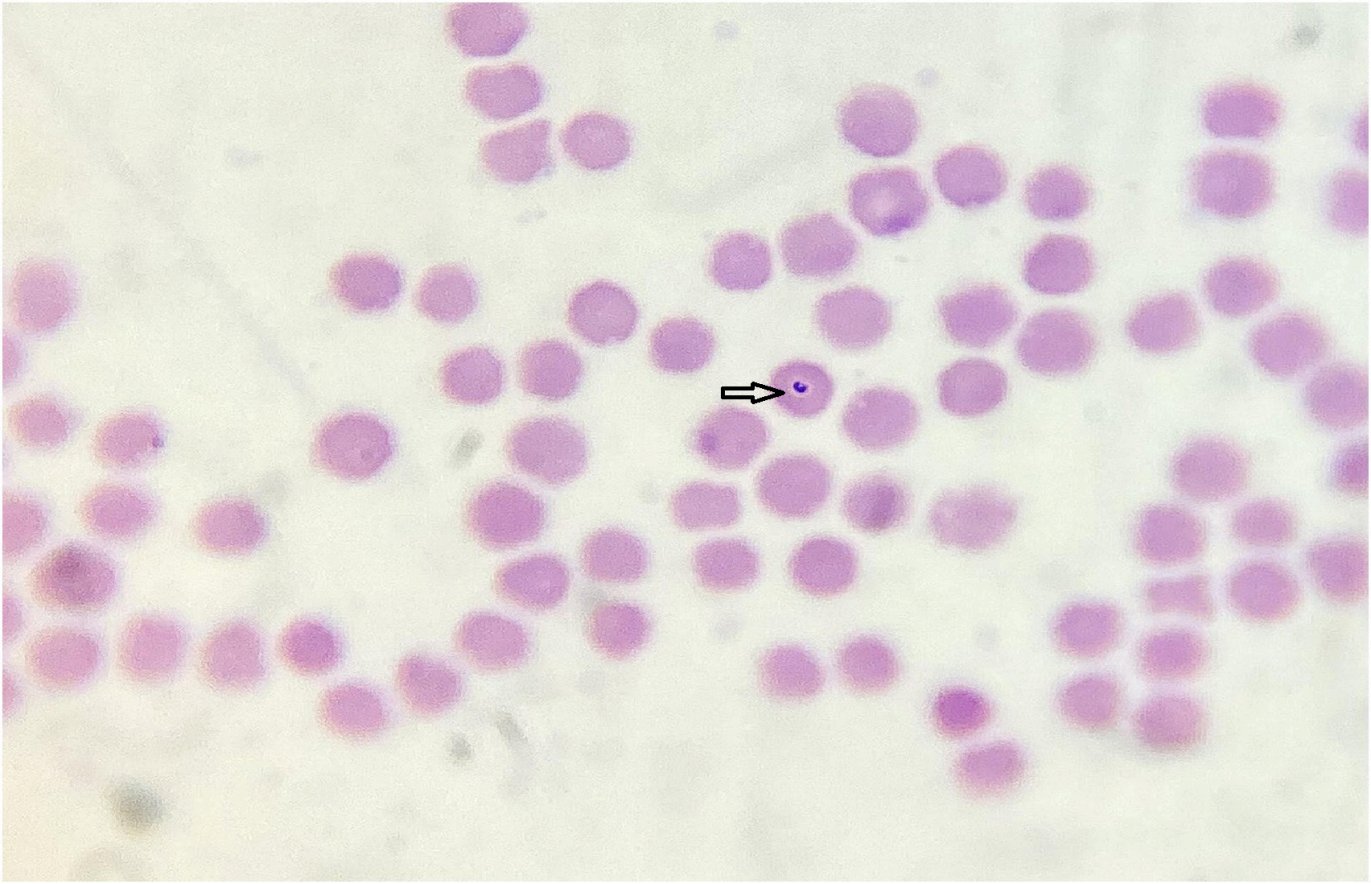
Typical form of *Theileria orientalis* in red blood cell (1,000x) (“Dr. Vaso Butozan”, 2024).

Microscopic examination of 35 blood samples revealed intracellular and extracellular bacteria in 31 samples. These bacteria were found in the erythrocytes, typically localized peripherally, and exhibited shapes ranging from spherical to coccobacillary (Figure 2). It was impossible to distinguish between the species within the genera *Anaplasma* and *Mycoplasma*. No bacterial structures were detected in white blood cells or platelets. Considering results, differential diagnostics were undertaken using molecular biology methods. Piroplasms were detected in 20 samples. The morphological analysis of merozoites and intraerythrocytic structures made it challenging to diagnose the species level accurately. However, the typical form of *T. orientalis* is presented in Figure 3.

### 3.2 Results of molecular detection of *Anaplasma phagocytophilum*

One sample from Gacko locality (Figure 1) tested positive for the presence of A. *phagocytophilum* using qPCR (Ct = 23.23). The positive dairy cow showed no signs of the disease and was 8 years old at the time of sampling.

### 3.3 Phylogenetic analysis of *A. phagocytophilum* sequence

**Figure 4:**
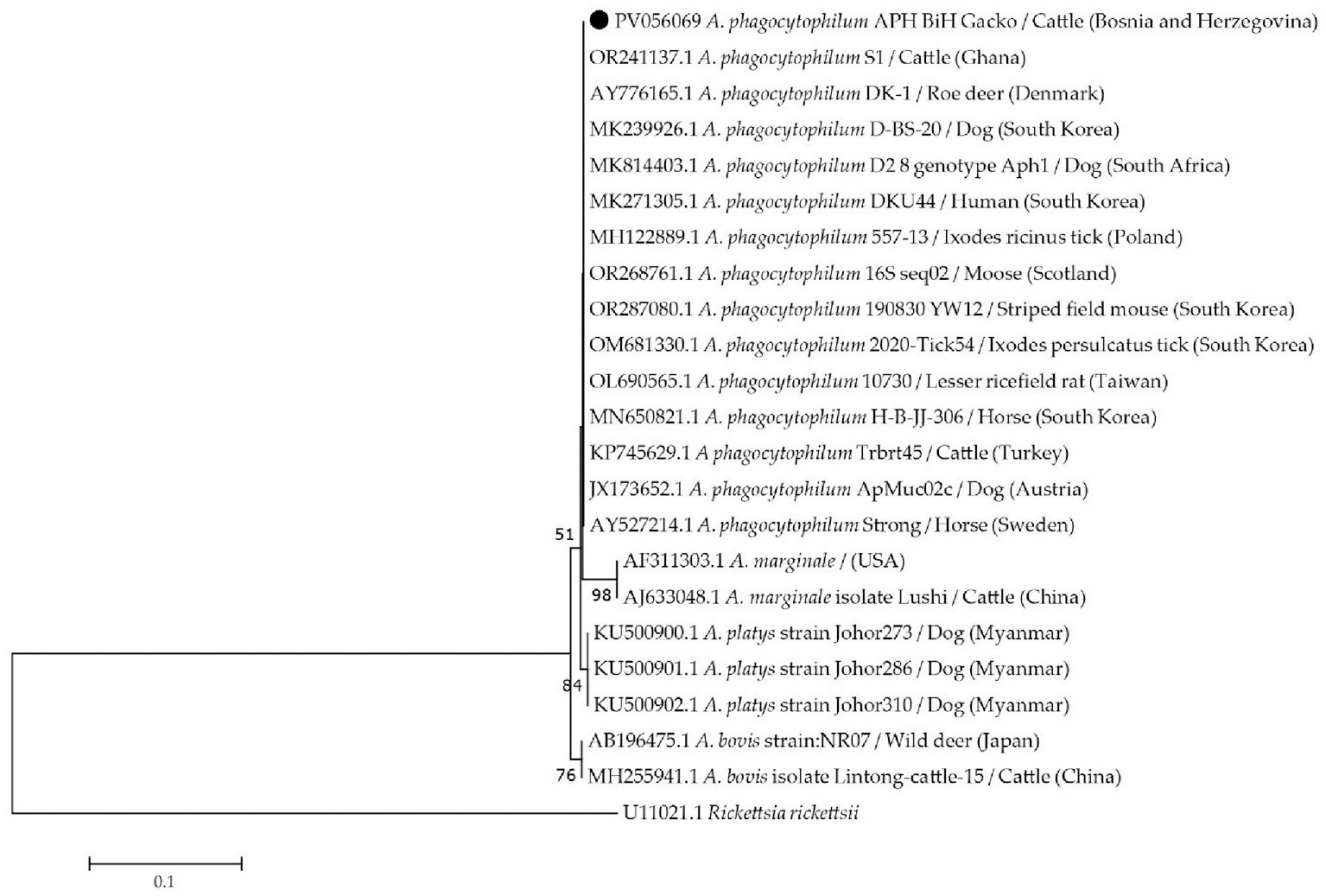
The Maximum likelihood phylogenetic tree based on the nucleotide sequences of the partial 16S rRNA gene of *Anaplasma* spp. *Rickettsia ricketsii* was included as outgroup. The numbers represent the percentage of 1,000 bootstrap iterations supporting the nodes and only percentages >50% are shown. Nucleotide sequence of *A. phagocytophilum* from this study is marked with a black circle. GenBank accession numbers, isolate names, hosts, and the countries of origin are indicated for each sequence.

**Figure 5:**
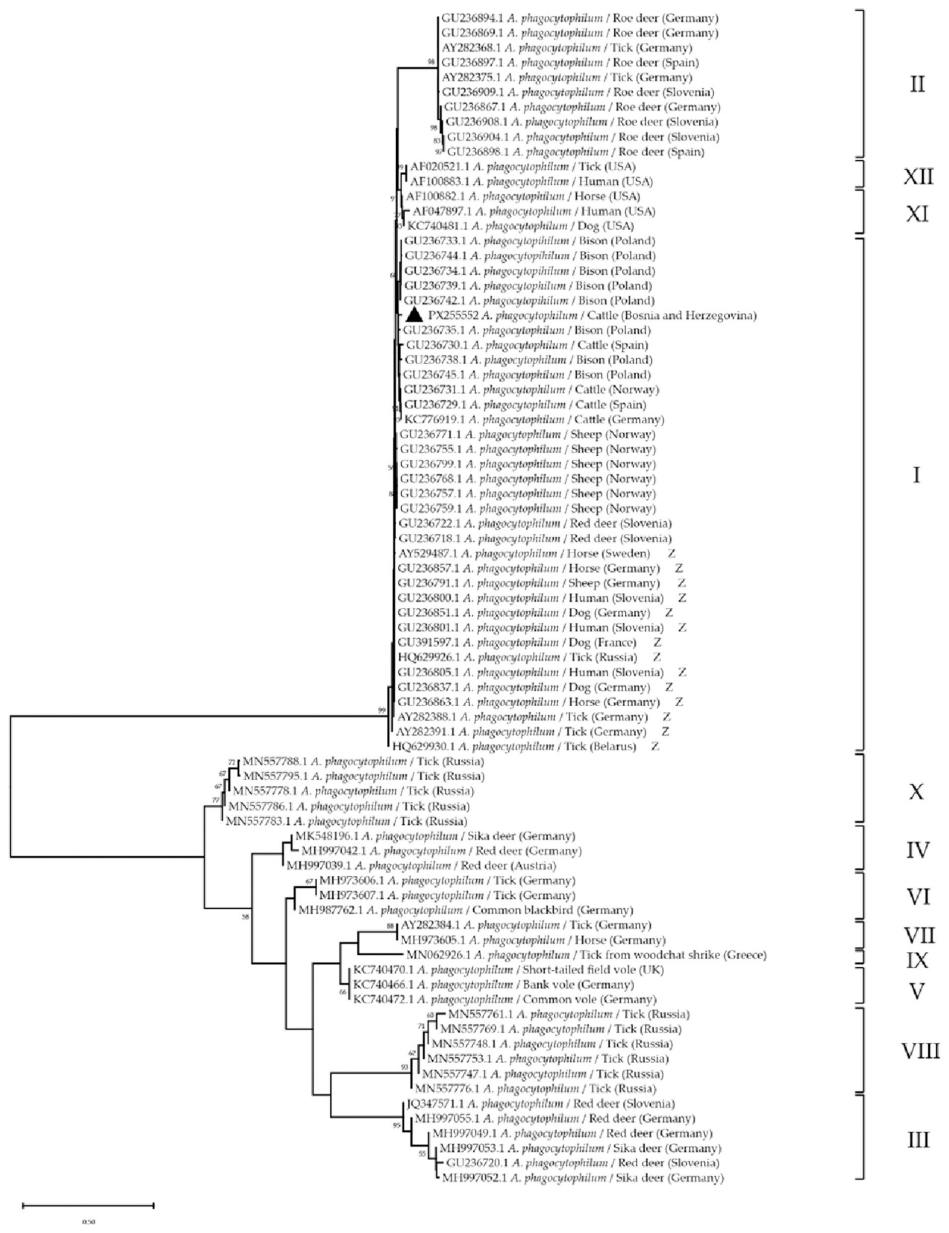
The Maximum Likelihood phylogenetic tree based on the nucleotide sequences of the partial *ankA* gene of *Anaplasma phagocytophilum*. The numbers represent the percentage of 1,000 bootstrap iterations supporting the nodes and only percentages >50% are shown. Nucleotide sequence of *A. phagocytophilum* from this study is marked with a black triangle. GenBank accession numbers, isolate names, hosts, and the countries of origin are indicated for each sequence. Sequences are grouped according to Rar et al. (39) Zoonotic strains from group I are marked with “***Z***”.

Phylogenetic analysis of the partial 16S rRNA gene performed by aligning the 21 *Anaplasma* spp. sequences with the sequence obtained in this study confirmed its grouping with *A. phagocytophilum* representatives (Figure 4) with the highest similarity with isolates from Ghana OR241137.1 (100%) and Brasil KP642755.1 (100%) and Denmark 253AJ776165.1 (99%). Maximum-likelihood analysis of the partial *ankA* gene placed our cattle isolate (PX255552) within group I according to Rar et al. (39), clustering with European ruminant isolates (e.g., bison/cattle from Central and Northern Europe), and distinct from the zoonotic group I subcluster (marked “*Z*” in Figure 5). The Bosnian sequence formed a well-supported branch with neighboring European ruminant strains, indicating affiliation with a ruminant-associated lineage rather than the zoonotic ecotype. This *ankA* placement complements the 16S rRNA result and refines the molecular characterization of the detected *A. phagocytophilum*, consistent with the 12-cluster *ankA* scheme used in our study.

### 3.4 Results of molecular detection of *Babesia*/*Theileria*

DNA fragment of *Babesia/Theileria* species was detected in 34 samples (29 from Gacko and 5 from Bileća). High quality amplicons (*n* = 5) were subjected to sequencing and phylogenetical analysis.

### 3.5 Phylogenetic analysis of *T. orientalis* sequences

**Figure 6:**
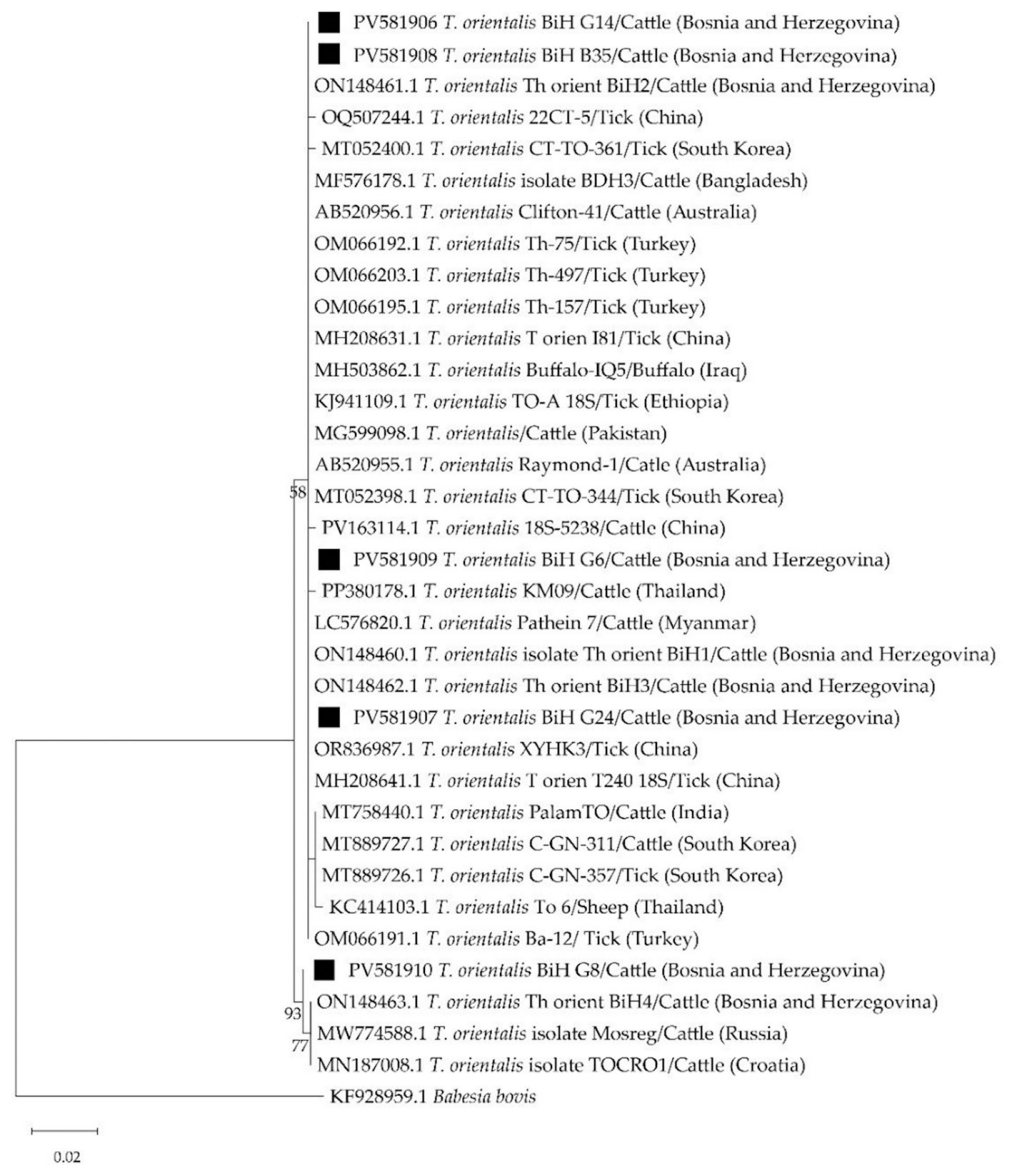
The Maximum Likelihood phylogenetic tree based on the nucleotide sequences of the partial 18S rRNA gene of *Theileria orientalis. Babesia bovis* was included as outgroup. The numbers represent the percentage of 1,000 bootstrap iterations supporting the nodes and only percentages >50% are shown. Nucleotide sequences of *T. orientalis* from this study are marked with black squares. GenBank accession numbers, isolate names, hosts, and the countries of origin are indicated for each sequence.

The phylogenetic tree based on the partial 18S rRNA gene sequences of *T. orientalis* shows limited genetic variation, consistent with the highly conserved nature of ribosomal RNA genes (Figure 6). Despite this, regional clustering is noticeable, especially among Bosnian isolates (PV581906–PV581909 and ON148460–ON148462), which form a distinct monophyletic group. The presence of related sequences from other parts of the world reinforces the idea of a globally distributed and genetically stable *T. orientalis* population. Although the 18S rRNA gene offers limited resolution for intra-species variation, the tree reflects subtle geographic structuring. PV581910 is part of a regional *T. orientalis* population spanning southeastern Bosnia and Croatia, with minimal sequence divergence, consistent with the conserved 18S gene and denoting stable endemic transmission cycles in the area. Nucleotide sequence identities among the Bosnian isolates from this study ranged from 99.3 to 100% (pairwise distance up to 0.7%), while comparison with other previously reported sequences from Bosnia and Herzegovina showed 99.1% to 100% similarity (pairwise distance up to 0.9%). When compared to sequences from other countries, the similarity ranged from 98.8 to 100% (pairwise distance up to 1.1%).

## 4 Discussion

*Theileria orientalis* is protozoan parasite that, in most cases, causes non-specific disease signs making it problematic to define its clinical significance. These piroplasms are genereally not eliminated from the circulation after infection, and parasitemia persists in infected animals for prolonged periods, even for life if carrier state is established (18). Our study confirms a high number of PCR positive piroplasmosis cases in grazing clinically healthy cattle from Romania region in Eastern Herzegovina. A case study from Croatia documented that *T. orientalis* contributed to the lethal outcome in three cows as a co-infection with *A. marginale* and *A. bovis* (32). Inconsistent with our findings, *T. orientalis* was recorded in asymptomatic cattle from neighboring Serbia with a very low prevalence (5/135, 3.70%) (11). It's increasingly detected in some European countries like Italy, Spain, Portugal, Greece, and the Balkans (25–28). Prevalence varies regionally but is rising due to climate change and animal movement. Although genotyping of the detected strains was not performed in this study, the molecular evidence confirms active circulation of *T.orientalis* in the region. The identification of the enzootic focus in southern part of the country suggests a stable transmission cycle likely supported by favorable environmental conditions and the presence of competent tick vectors, such as *Haemaphysalis* spp. and *Rhipicephalus* spp. (49). The high percentage of infected asymptomatic cattle in these areas could be an indicator of the endemic stability of oriental theileriosis, or the relatively low pathogenicity of the *T. orientalis* genotypes present (17, 25). This study was geographically limited to selected farms in the Herzegovina region, which may not fully represent the spatial extent of the enzootic focus and the lack of direct tick collection and identification limited our ability to confirm the local vector species responsible for transmission. Animal movement, pasture sharing and tick control practices are not considered, further limiting the ability to assess risk factors for enzootic establishment.

This study also documents the presence of *A. phagocytophilum*-specific DNA in a cattle blood sample, confirming its first detection in these animals in Eastern Herzegovina (Romanija region). These results align with previously published results of *A. phagocytophilum* presence in ticks (7) and dogs (8, 9) and extend current knowledge on its geographical prevalence within different hosts, also confirming its ongoing circulation.

The obtained nucleotide sequence clustered with sequences from Austria, Poland, Denmark, and Türkiye suggesting a potential epidemiological link and pathogen circulation across these regions. The observed clustering of 16S rRNA sequences suggests a stable *A. phagocytophilum* lineage circulating across Europe, extending through the Balkans, possibly maintained by wildlife reservoirs and tick migration rather than livestock trade. The detection of *A. phagocytophilum* in wild and domestic ruminants, especially sheep and goats, has been performed throughout Europe with varying reports concerning prevalence (4, 50–52). A recent study from Poland performed on several wild cervid species indicated the potential reservoir status of these animals (53). Interestingly, another Polish investigation (54) of free-grazing goats in forested regions found no evidence of *A. phagocytophilum*, suggesting this species has lower infection rates compared to other ruminants, consistent with the claims from a German study by Rubel et al. (55).

Available data from Germany indicates notable prevalence and confirms the circulation of numerous strains of *A. phagocytophilum* in dairy cattle, likely sustained by wildlife reservoirs (4). Also, in Türkiye, Aktas et al. (56) detected *A. phagocytophilum* in small ruminants, although a genetically distinct variant (*A. phagocytophilum*-like 1) was notably more prevalent, suggesting that the existence of these regionally adapted genetic variants warrants further examinations. Comparable to our examination, the *msp2* gene was also used as a target for *A. phagocytophilum* detection in a study from Great Britain that confirmed its presence in cattle, sheep, deer, and *I. ricinus* ticks, and the potential role of cattle in the pathogen's epidemiological cycle was proposed (57).

The *ankA*-based phylogeny refines the molecular characterization of our strain. Rar et al. (39) showed that *ankA* diversity resolves *A. phagocytophilum* into 12 host/vector-associated clusters in agreement with MLST and *groEL* ecotypes. The sample from this study grouped within *ankA* cluster I. However it lies outside the zoonotic subcluster, paralleling reports of ruminant-restricted lineages circulating in Central and Northern Europe (4, 51). In Poland, cervids act as key reservoirs and harbor diverse *groEL* genotypes, and recent roe/red deer typing shows *ankA*/*groEL* combinations that include group I variants but also cervid-adapted lineages (53, 58). Wild boar have also carried human-pathogenic variants in Poland, underscoring a broader wildlife reservoir (59). Ecotype analyses from Great Britain further highlight that cattle infections often align with *groEL* Ecotype I, while Turkey has documented confirmed bovine cases without *ankA* typing (57, 60). Collectively, these data support our interpretation that the Bosnian isolate belongs to a ruminant-associated *ankA* group I lineage maintained primarily in a wildlife–*I. ricinus* cycle rather than through cattle-to-cattle transmission (61).

The diagnosis of anaplasmosis in cattle commonly relies on observation of clinical symptoms, followed by the examination of Giemsa-stained thin blood smears that enable the inclusion body identification. In our study, the spherical and coccobacillary bacteria were located within erythrocytes, primarily at the cell margins. However, the observation of *Anaplasma* and *Mycoplasma* species in microscopic examination seen in this study needed further evidence. Since there is a possibility that the observed changes in coloration in the blood smears might be due to stain residue or Howell-Jolly bodies, Heinz bodies, or Pappenheimer bodies. Our research did not confirm the presence of *A. marginale*, which had been reported in symptomatic cattle in neighboring Croatia (32). Additionally, microscopy yielded no bacterial structures in white blood cells or platelets. It is well known that *A. phagocytophilum* DNA is detectable days before visible inclusions can be identified through blood smear microscopy, and the proportion of infected leukocytes can vary considerably (2). The qPCR protocol (*msp2* gene) that was used in this study was shown to have high specificity and sensitivity, so the detection of *A. phagocytophilum* DNA in one sample can be regarded within the limits of qPCR method.

Limitation of this study includes small sample size (*n* = 35) which might be reason for detection of only one positive sample for *A. phagocytophilum*. At this time, it was not possible to conduct large-scale research which is necessary to provide further insight into the spread and clinical significance of this tick-borne pathogen occurring in cattle. This study was conducted as an exploratory investigation rather than a prevalence study, with selective sampling guided by veterinary reports of tick infestations. Our results because of this limitation can not be extrapolated to the whole population of cattle of Bosnia and Herzegovina. Further limitation of the study is the lack of data on vector species ticks, which were not collected at this point because of the focus on animal health.

Different *Anaplasma* species have various transmission vectors: *A. ovis* and *A. marginale* are mainly spread by *Rhipicephalus* and *Dermacentor* ticks, *A. phagocytophilum* is transmitted via *Ixodes* species, while *A. platys* is spread by *R. sanguineus*, the brown dog tick (62–64). In Bosnia and Herzegovina, eleven species of hard ticks from the *Ixodidae* family have been documented, with *I. ricinus* being the most prevalent, followed by *D. marginatus, R. bursa, Hyalomma marginatum, R. sanguineus* sensu lato, *Haemaphysalis punctata, I. canisuga, D. reticulatus, I. hexagonus*, and *H. concinna* (65). A study by Stevanović et al. (66) validated that *R. bursa* dominates among the tick species parasitizing sheep in Eastern Herzegovina. However, this study failed to collect ticks parasitizing cattle, leading to uncertainty regarding the tick species composition, which can only be inferred from epidemiological data. Furthermore, the seasonal occurrence of the diseases caused by tick-borne pathogens was not considered since sampling was performed in a snapshot study in cattle based on epidemiological data.

Differently from certain *Anaplasma* species, there is no evidence that *A. phagocytophilum* undergoes transovarial transmission within *Ixodes* ticks. Accordingly, a reservoir host is essential for maintaining its endemic life cycle in the environment (1). Rubel et al. (55) reported a strong correlation between the presence of cats and dogs on livestock farms with exposure to *Anaplasma* spp. in farmed ruminants. However, their role as reservoir hosts of *A. phagocytophilum* is controversial and highly unlikely (67). High prevalence rates of *A. phagocytophilum* detected in wild ruminant populations in Italy highlight their importance as hosts in the infectious cycle of this pathogen (51). Additionally, wild ruminants not only aid in the development and reproduction of *I. ricinus* ticks but also harbor various *A. phagocytophilum* genetic variants. Nevertheless, at present, available information regarding reservoir hosts of *A. phagocytophilum* in Eastern Herzegovina remains insufficient, and the specific host species involved in its transmission cycle remains to be determined.

## 5 Conclusions

This study confirms the presence evidence suggestive of an enzootic focus of *T. orientalis* in Herzegovina, which is driven by subclinical infections. This suggests possible endemic stability and a dynamic equilibrium in the interactions among hosts, vectors and pathogens. The high number of *Babesia/Theileria*-positive cattle observed in this study supports the notion that oriental theileriosis is widespread in Bosnia and Herzegovina. Microscopic diagnostics appear to have limitations when compared to PCR diagnostics for detecting piroplasms in cattle.

In addition, we provide the first molecular evidence of *A. phagocytophilum* DNA in cattle from Herzegovina. The *ankA* gene analysis further indicates that this Bosnian isolate belongs to a ruminant-associated group I lineage distinct from the zoonotic subcluster, underscoring the need to monitor wildlife–*I. ricinus* cycles when assessing potential spillover to cattle and humans.

## Data Availability

The datasets presented in this study can be found in online repositories. The names of the repository/repositories and accession number(s) can be found in the article/supplementary material.
